# Optimizing a Digital Mindfulness‐Based Intervention to Reduce Risk Factors for Disordered Eating: A Multiphase Optimization Strategy (MOST) Feasibility Trial

**DOI:** 10.1002/eat.24546

**Published:** 2025-09-16

**Authors:** Emma L. Osborne, Ben Ainsworth, Nic Hooper, Paul Chadwick, Melissa J. Atkinson

**Affiliations:** ^1^ Department of Psychology University of Bath Bath UK; ^2^ School of Psychology, Faculty of Environmental and Life Sciences University of Southampton Southampton UK; ^3^ Enable Inclusion Trust South Gloucestershire UK

**Keywords:** body image, digital intervention, disordered eating, emotion regulation, factorial trial, mindfulness‐based intervention, multiphase optimisation strategy, negative affect, self‐compassion, weight and shape concerns

## Abstract

**Objective:**

Mindfulness‐based interventions (MBIs) show promise in reducing risk factors for disordered eating. Digital delivery offers scalable reach, but effects vary and engagement is generally suboptimal—potentially due to the length, complexity, and variation in content. Identifying the most effective and engaging components is necessary to streamline interventions. Guided by the multiphase optimization strategy (MOST), this study assessed the feasibility of a factorial trial to evaluate whether including emotion regulation (ER) and self‐compassion (SC) enhances the effects of decentred awareness and acceptance (DA + A, observing thoughts and feelings as separate from the self) in a digital MBI.

**Method:**

A 2 × 2 factorial design tested four intervention conditions. Each included DA + A content, with or without added ER and/or SC. Participants (*N* = 92, *M* = 21.1 years, range = 18–32) were randomized and given 3‐weeks' access. Feasibility outcomes included recruitment, retention, data analysis, engagement, and acceptability. Intervention outcomes—weight and shape concerns, negative affect, mindfulness, ER, and SC—were assessed at baseline, postintervention, and 1‐month follow‐up.

**Results:**

Recruitment exceeded targets. Retention was acceptable (58% completed postintervention and 59% follow‐up assessments). All planned analyses for the main trial were feasible. Engagement met preregistered criteria (77%–85% completed Module 1 across ER and SC conditions; mean of four modules). Acceptability was high (*M* = 4.28/5). Participants valued reminders and provided design suggestions.

**Discussion:**

Findings support progression to a fully powered optimization trial. This study demonstrates that MOST can be embedded within a pilot study, providing a foundation to develop more effective, engaging, and scalable interventions.

**Summary**

- Factorial feasibility trial of a digital mindfulness‐based intervention for disordered eating risk factors, guided by the multiphase optimization strategy (MOST).
- Participants received training in decentered awareness and acceptance, with or without added emotion regulation and self‐compassion components.
- Feasibility outcomes across recruitment, retention, data analysis, engagement, and acceptability support progression to a fully powered optimization trial.
- Demonstrates that MOST can be applied to pilot studies to provide a strong foundation for developing effective, engaging, and scalable interventions.

## 1 Introduction

### 1.1 Background and Rationale

Disordered eating refers to maladaptive behaviors (e.g., fasting, purging, binge eating) and related cognitions (e.g., body image disturbance), regardless of whether clinical criteria for an eating disorder are met. Even at low levels, it is associated with significant consequences, including impaired quality of life, psychological distress, anxiety, depression, and self‐harm (Bornioli et al. 2019; Kärkkäinen et al. 2018; Micali et al. 2017; Wade et al. 2012). UK estimates indicate that 32.7% of females and 7.6% of males reported disordered eating in the past year at age 16, increasing to 36.9% and 19.2% by age 24 (Warne et al. 2021). A wide range of risk factors are associated with disordered eating, spanning sociocultural, biological/genetic, and psychological domains (Barakat et al. 2023). Interventions targeting key modifiable risk factors (e.g., thin‐ideal internalization, pressure to be thin, weight and shape concerns, dieting, and negative affect) have the potential to reduce the experience, onset, and burden of disordered eating. Among the most robust of these are weight and shape concerns (i.e., body dissatisfaction, preoccupation, and overvaluation in self‐judgment) and negative affect (Jacobi and Fittig 2010; Pennesi and Wade 2016).

Mindfulness‐based interventions (MBIs) may address these risk factors by promoting more adaptive responses to negative experiences—for example, by encouraging a noncritical self‐view, reducing automatic maladaptive reactions, decreasing experiential avoidance, and thereby supporting more adaptive emotion regulation (ER) (Atkinson 2015). MBIs have shown promise in reducing weight and shape concerns and negative affect (Beccia et al. 2018; Linardon, Gleeson, et al. 2019), and their digital delivery (i.e., via smartphone apps and web‐based platforms) enables scalable reach (Linardon, Cuijpers, et al. 2019; Mrazek et al. 2019). However, effects vary considerably, and user engagement—whether measured by uptake, time spent, number of visits, behavioral adoption, or completion rates—is generally suboptimal; for example, up to 55% of participants in some trials failed to access their prescribed mindfulness app (Linardon 2023). This may reflect the length, complexity, and variability of program content. Identifying the most effective and engaging components of MBIs may enhance both outcomes and engagement by streamlining programs to focus on what matters most (Watkins and Newbold 2020).

MBIs teach individuals to become more aware of their moment‐by‐moment experiences, to relate to them from a decentred perspective (i.e., by seeing thoughts and feelings as temporary mental events, separate from the self, and not necessarily true), and to accept these experiences as they are (Bishop et al. 2004). This combination of decentred awareness and acceptance of present‐moment experience (DA + A) is consistently emphasized in theoretical models of mindfulness and recognized as a crucial mechanism of change (Bishop et al. 2004; Shapiro et al. 2006; Teasdale et al. 2002), and is a core component of widely used MBIs (e.g., Kabat‐Zinn 1990; Segal et al. 2002). It can, therefore, be considered foundational to MBIs.

However, many MBIs also include additional components intended to enhance effectiveness, such as stress management, cognitive restructuring, ER, distress tolerance, and self‐compassion (SC) (see, e.g., Supporting Information Data S1). This is also true of MBIs targeting disordered eating risk factors, which often incorporate components such as SC, gratitude, ER, and healthy lifestyle‐related content (e.g., Albertson et al. 2015; Atkinson and Wade 2015, 2016; Fuller‐Tyszkiewicz et al. 2019; Rodgers et al. 2018). For example, *The Mindfulness Mode*, a facilitator‐led MBI, has demonstrated reductions in weight and shape concerns, negative affect, and other risk factors in adolescent girls and young women (Atkinson and Wade 2015, 2016), and included content targeting ER and SC—but did not systematically test whether these elements were necessary for the observed effects.

Despite longstanding calls to identify the mechanisms and active components of psychological interventions (Ainsworth et al. 2023; Holmes et al. 2018; Kazdin 2007), we still do not fully understand how MBIs work—that is, which components are most effective and whether they engage hypothesized psychological mechanisms (Stein and Witkiewitz 2020). Some of these added components may offer benefits, but others may have minimal impact, dilute, or distract from core concepts, or have unintended iatrogenic effects. This is problematic not only in terms of effectiveness but also engagement, as additional content often increases the time and effort required from users. This added burden may contribute to dropout, meaning users miss out on potential helpful content (Fitzsimmons‐Craft et al. 2024). This is especially relevant for digital programs targeting eating disorder risk factors, where motivation may be lower and engagement is often suboptimal (Linardon et al. 2020).

To address these challenges, there is growing interest in optimizing interventions using approaches such as the multiphase optimization strategy (MOST; Collins 2018; Collins et al. 2024). MOST involves selecting promising intervention components (“candidates”) and systematically evaluating their individual effects through an *optimization* randomized controlled trial (ORCT), such as a factorial experiment. Once the most effective and practical combination of components is identified, the resulting intervention can be tested against an appropriate control in an *evaluation* randomized controlled trial (ERCT). This approach enables researchers to determine which components are essential and which can be removed, guiding the development of more effective, efficient, and scalable interventions. MOST is, particularly, well‐suited to complex, multicomponent interventions like MBIs (Garland and Howard 2018) and has the potential to significantly advance eating disorder research (Manasse et al. 2019), yet its application to these fields remains limited.

Two components with potential to improve outcomes beyond foundational DA + A content are ER and SC. ER refers to the ability to understand, accept, and manage emotions while acting effectively during distress (Gratz and Roemer 2004). SC involves treating oneself with kindness, recognizing struggles as part of the human experience, and maintaining mindful awareness without overidentification (Neff 2023). As widely recognized mechanisms underlying the effects of MBIs (Baer 2010; Chiesa et al. 2014; Hölzel et al. 2011; Neff and Dahm 2015), these components are included in the most widely used MBIs (e.g., *Mindfulness‐Based Cognitive Therapy*, Segal et al. 2002; *Dialectical Behavior Therapy*, Linehan 1993; *Mindful Self‐Compassion*, Neff and Germer 2013), as well as in those adapted for eating and body image concerns (e.g., *Mindfulness‐Based Eating Awareness Training*, Kristeller and Wolever 2010; *The Mindfulness Mode*, Atkinson and Wade 2015, 2016; see Supporting Information Data S1).

Both constructs are also relevant to disordered eating. ER difficulties are central in theoretical models (Pennesi and Wade 2016), and longitudinal evidence supports its role in mediating the effects of mindfulness on key risk factors (Osborne et al. 2022). Improvements in ER may increase emotional awareness, reduce avoidance, and support more flexible coping with intense emotions, thereby helping individuals manage experiences associated with increased risk of disordered eating (Teper et al. 2013; Chambers et al. 2009; Roemer et al. 2015). Similarly, higher SC predicts decreases in weight and shape overvaluation (Linardon and Messer 2023), and improvements in SC have been shown to mediate reductions in binge eating following an MBI (Pinto‐Gouveia et al. 2016). Improvements in SC may reduce the impact of body image disturbance by encouraging a noncritical self‐view and fostering kindness toward one's body and appearance (Atkinson 2015; Vanzhula and Levinson 2020).

### 1.2 The Current Study

This feasibility trial, conducted during the preparation phase of MOST, precedes a fully powered 2 × 2 factorial ORCT to optimize a digital MBI targeting risk factors for disordered eating. It tested a digital, program‐led version of *The Mindfulness Mode* (Osborne, Ainsworth, Hooper, Chadwick, Atkinson 2025a). Given that DA + A can be considered foundational to MBIs and provides the experiential context in which components such as ER and SC are typically introduced and applied, it was included in all conditions as a “minimal necessary” intervention (Manasse et al. 2019). While the main trial will evaluate whether ER and SC components enhance the effects of DA + A, the present feasibility study aimed to reduce uncertainty around the evaluation and intervention designs to determine whether and how to proceed to a full‐scale ORCT, consistent with guidance for complex intervention research (Skivington et al. 2021). Its primary objectives aligned with key feasibility uncertainties (see Table 1).

**TABLE 1:** Key uncertainties, corresponding primary objectives, and assessment methods.

Key uncertainty	Primary objective	Method of assessment
*Feasibility of the evaluation design*		
Recruitment	Assess ability to recruit participants within timeframe.	Number recruited during the recruitment period and recruitment sources reported by participants.
Retention	Assess retention at each timepoint.	Proportion of randomized participants with outcome data at post and follow‐up.
Data analysis	Assess viability of main trial analyses.	Collection of all data required to support the main trial and ability to run the intended analyses.
*Feasibility of the intervention design*		
Engagement	Assess level of intervention engagement.	(i) Average number of modules completed; (ii) Proportion of participants engaging in “essential use” (completion of at least Module 1).
Acceptability	Assess intervention acceptability.	Participant responses to the Theoretical Framework of Acceptability questionnaire and open‐ended feedback.

## 2 Method

### 2.1 Study Design

We used a 2 (ER: on, off) × 2 (SC: on, off) factorial design, with assessments at three time points: baseline, postintervention (3 weeks), and 1‐month follow‐up. The study protocol was preregistered on the Open Science Framework (Osborne, Ainsworth, Hooper, Chadwick, Atkinson 2025). Participants were randomized to one of four conditions representing all combinations of the ER and SC components, each delivered alongside a constant component focused on DA + A (see Table 2). Randomization was implemented via LifeGuide+'s automated function using a probability‐based algorithm (0.95 certainty) to maintain approximate 1:1:1:1 group balance.

**TABLE 2:** Experimental conditions in the 2 × 2 factorial design.

Experimental condition	Intervention condition	Intervention components		
		DA + A	ER	SC
1	DA + A + ER + SC	On	On	On
2	DA + A + ER	On	On	Off
3	DA + A + SC	On	Off	On
4	DA + A only	On	Off	Off

*Note*: All participants received DA + A (decentered awareness and acceptance of present‐moment experience) as a constant component. Participants were randomized to one of four conditions representing all combinations of emotion regulation (ER) and self‐compassion (SC).

Abbreviations: Off = component not included; On = component included.

### 2.2 Sample Size

We used a formal framework for hypothesis testing of process outcomes in pilot trials (e.g., retention, engagement) to determine the planned sample size. Our estimate was based on a one‐sided hypothesis test with *α* = 0.05, power = 0.80, and cut‐off differences defined by two‐tier progression criteria, mapped onto red (unacceptable) and green (acceptable) zones: (i) retention of ≥ 75% at postintervention, with ≤ 50% indicating a main trial would not be feasible and (ii) engagement of ≥ 50% of participants in at least “essential use” of the intervention, with ≤ 25% indicating a main trial would not be feasible. These thresholds reflected a 25% difference between unacceptable and acceptable outcomes, consistent with guidance for planning progression criteria in pilot trials (Lewis et al. 2021). “Essential use” was defined as completion of at least Module 1, informed by findings from Ainsworth et al. (2017), which suggest that digital interventions may have their greatest impact during the first session. Module 1 was intentionally designed to provide immediate benefit by introducing key concepts across the assigned component(s) and setting the stage for the rest of the program. Using the look‐up grid for these cut‐points, the required sample sizes were: (i) 27 participants overall for retention (a study‐wide outcome) and (ii) 25 participants per level of each component for engagement (total = 50). Allowing for a 37% study attrition rate, typical of fully automated digital mental well‐being intervention trials (Groot et al. 2023), we aimed to recruit 80 participants.

### 2.3 Participants

We recruited young people aged 16–35 through university and community channels, including email lists, online advertisements, and posters on campus and in local cafés. This age range was targeted because older adolescence and young adulthood represent vulnerable periods for the development and experience of disordered eating (Nagl et al. 2016).

### 2.4 Procedure

Advertisements promoted a study evaluating an online mindfulness program designed to improve body image and mood. These directed individuals to LifeGuide+ (https://lifeguideonline.org/), a digital intervention platform developed by the University of Southampton, where they could access further information, confirm their age, and provide informed consent. Participants completed baseline questionnaires (demographics and outcome measures) before randomization and were given access to the intervention. They completed a postintervention questionnaire after 3 weeks (acceptability and outcomes) and a follow‐up questionnaire (outcomes only) 1 month later. Participants received up to three automated email reminders to complete each of the postintervention and follow‐up questionnaires and were given a £10 Amazon e‐voucher for each of these completed assessments. The University of Bath Research Ethics Committee approved the study (6813–9587).

### 2.5 Intervention

We adapted an existing evidence‐based program, *The Mindfulness Mode* (Atkinson and Wade 2015, 2016), by separating out its components for experimental evaluation and converting it into a digital format. This intervention was selected because it is a multicomponent MBI specifically designed to address body image and sociocultural appearance pressures, with demonstrated effects on disordered eating risk factors in adolescent girls and young women, and includes content related to ER and SC.

The adapted intervention, *eMindfulness Mode*, is a digital, program‐led version, meaning it is delivered by the program rather than a facilitator (Fairburn and Patel 2017). We used the person‐based approach (PBA), an internationally recognized iterative method to optimize intervention acceptability, to guide adaptation and ensure relevance to the target population (Yardley et al. 2015). The development process and final intervention are described in detail elsewhere (Osborne, Ainsworth, Hooper, Chadwick, Atkinson 2025a) and summarized here using the TIDieR checklist (Hoffmann et al. 2014; Supporting Information Data S2). *eMindfulness Mode* is a mobile‐friendly website comprising an introduction and nine modules, each lasting approximately 10–15 min (with Module 1 taking around 15–20 min, as it introduces the core concepts of the program).

To fit the 2 × 2 factorial design, we created four conditions by explicitly integrating ER and/or SC into all the modules in the relevant conditions, including Module 1, and omitting them from others. These adaptations drew on the work of Paul Gilbert and Kristin Neff, early iterations of the *Overcoming Perfectionism* program (Shafran et al. 2018), and the MANTRA model (Schmidt and Treasure 2006). They were implemented by adjusting the framing of DA + A, clarifying application contexts, and increasing emphasis on the intended components. A detailed overview of module themes, topics, and activities by condition, along with example intervention screenshots, is provided in Supporting Information Data S3.

All conditions included DA + A, which encouraged present‐moment awareness and acceptance of unwanted body‐related experiences to reduce their impact. Each condition featured experiential exercises, framed body‐related thoughts and emotions as transient and separate from the self, and promoted nonjudgment and acceptance of these experiences. Two conditions included ER, covering common body‐related emotions (e.g., shame, guilt), typical coping strategies (e.g., suppression, rumination), and their costs. DA + A was positioned as a longer‐term alternative, and exercises targeted emotionally difficult situations or those where individuals might otherwise revert to less helpful coping strategies. Two conditions included SC, which built on DA + A to support self‐kindness in response to appearance‐related distress. These modules focused on recognizing the inner critic, responding with compassion, and practicing SC in daily life (e.g., during social comparisons or media exposure).

Participants were encouraged to work through the program over 3 weeks and practice activities between modules. Participants in conditions with added components had access to more content within the modules. To support engagement, three standardized reminder emails were sent manually on a fixed schedule. While the content was fixed, the program allowed some self‐tailoring (e.g., audio/text options, personalized schedules, written reflections). Participants logged in with individual usernames and passwords, enabling progress tracking, and could contact the research team with questions about the program or their participation.

## 3 Measures

### 3.1 Demographics

Participants reported their age, gender, ethnicity, postcode, and education level.

### 3.2 Feasibility Outcomes

These align with the primary feasibility objectives outlined in Table 1.

#### 3.2.1 Feasibility of the Evaluation Design

##### 3.2.1.1 Recruitment

We tracked the number of participants recruited over time, and at baseline, participants answered an open‐ended question on how they heard about the study.

##### 3.2.1.2 Retention

Defined as the proportion of randomized participants who completed assessments at postintervention and follow‐up.

##### 3.2.1.3 Data Analysis

We assessed whether the data collected could address the primary objectives of the main trial (see Box 1), using the measures described in Table 3.

**BOX 1:** Primary objectives of the main trial.

Evaluate the effectiveness of each additional component in reducing risk factors for disordered eating. Assess whether each additional component improves its targeted mechanism. Explore whether: Improvements in targeted mechanisms are associated with reductions in risk factors. Baseline levels of targeted mechanisms and risk factors influence the effectiveness of each component.

**TABLE 3:** Measures used to assess the primary objectives of the main trial.

Variable	Measure (example item, response options)	Reliability (*α*)
Weight and shape concerns	Eating Disorder Examination–Questionnaire (Fairburn and Beglin 1994), Weight and Shape Concern subscales, 12 items (e.g., “Have you had a strong desire to lose weight?”), rated 0 (*not at all*) to 6 (*markedly*). Higher mean scores reflect greater concerns. Instructions referred to the past 14 days (vs. original 28) to capture changes during the intervention, consistent with prior work (e.g., Sala et al. 2023).	0.92
Negative affect	Positive and Negative Affect Schedule (Watson et al. 1988), Negative Affect subscale, 10 items (e.g., “guilty”), rated 1 (ve*ry slightly or not at all*) to 5 (*extremely*), based on the past 14 days. Higher sum scores reflect greater negative affect.	0.83
Mindfulness	Five Facet Mindfulness Questionnaire–Short Form (Baer et al. 2012), 15 items (e.g., “I find myself doing things without paying attention”), rated 1 (*never or very rarely true*) to 5 (*very often or always true*). Higher sum scores reflect greater mindfulness.	0.65
Emotion regulation	Difficulties in Emotion Regulation Scale–Short Form (Kaufman et al. 2016), 18 items (e.g., “I am confused about how I feel”), rated 1 (*almost never*) to 5 (*almost always*). Higher mean scores reflect greater difficulties.	0.91
Self‐compassion	Self‐Compassion Scale–Short Form (Raes et al. 2011), 12 items (e.g., “I'm disapproving and judgmental about my own flaws and inadequacies”), rated 1 (*almost never*) to 5 (*almost always*). Higher mean scores reflect greater self‐compassion.	0.78

*Note*: Cronbach's *α* values were calculated using the full baseline sample (*N* = 92).

#### 3.2.2 Feasibility of the Intervention Design

##### 3.2.2.1 Engagement

Assessed via (1) the average proportion of modules completed by randomized participants and (2) the proportion who engaged in essential use.

##### 3.2.2.2 Acceptability

At postintervention, participants completed the theoretical framework of acceptability (TFA) questionnaire (Sekhon et al. 2022). Five‐point scales (see Table 8 note for anchors) assessed affective attitude, burden, ethicality, intervention coherence, opportunity costs, perceived effectiveness, self‐efficacy, and general acceptability, with an open‐ended question for additional feedback.

## 4 Data Analysis

Descriptive statistics were used to summarize recruitment, retention, engagement, and acceptability. Open‐ended responses were summarized descriptively. To assess the feasibility of main trial analyses, we used complete‐case data to explore whether the primary objectives outlined in Box 1 could be addressed. Paired‐samples *t* tests summarized overall changes from baseline to postintervention and follow‐up, across all conditions. ANCOVAs compared postintervention outcomes between participants who received versus did not receive each component, controlling for baseline scores. Pearson correlations examined associations between change scores in mechanisms and outcomes across all conditions. Moderation analyses were not conducted due to limited sample size. These were considered appropriate exploratory analyses for this feasibility study.

## 5 Results

### 5.1 Sample Characteristics

The mean age of the sample (*N* = 92) was 21.1 years (SD = 3.0; range = 18–32). No participants aged 16–17 were recruited, as the target sample size was met through initial recruitment methods primarily involving university channels. Most participants identified as women (93%) and as White (52%) or Asian/Asian British (33%). Educational attainment was high: 92% had completed A Levels or higher (59% A Levels, 25% undergraduate degree, 8% master's degree). Postcodes were available for 77 participants (84%), of whom 66% (51/77) were from areas of low deprivation (index of multiple deprivation [IMD] deciles 8–10). The mean IMD decile was 8.5 (SD = 2.4), indicating low overall deprivation. A full breakdown of ethnicity, education, and IMD is provided in Supporting Information Data S4.

Baseline scores for all outcomes and targeted mechanisms are presented in Table 4. Weight and shape concerns were elevated (*M* = 3.14, SD = 1.33), exceeding normative data reported for a nonclinical UK sample of young adults (weight concern: *M* = 1.94, SD = 1.57; shape concern: *M* = 2.34, SD = 1.62; Carey et al. 2019). Over three‐quarters of participants (71/92; 77%) scored above the combined normative mean for weight and shape concerns. Negative affect (*M* = 22.18, SD = 6.76) was also elevated relative to the UK adult norm (*M* = 16.00, SD = 5.90; Crawford and Henry 2004).

**TABLE 4:** Baseline scores for all outcomes and targeted mechanisms (*n* = 92).

Measure	*M* (SD)
Weight and shape concerns	3.14 (1.33)
Negative affect	22.18 (6.76)
Mindfulness	46.25 (6.94)
Emotion regulation difficulties	2.68 (0.72)
Self‐compassion	2.72 (0.60)

### 5.2 Feasibility Outcomes

#### 5.2.1 Feasibility of the Evaluation Design

##### 5.2.1.1 Recruitment

Ninety‐two participants were recruited and completed the baseline questionnaire between March 4 and March 21, 2025. Participants were randomized to one of four intervention conditions: DA + A + ER + SC (*n* = 24), DA + A + ER (*n* = 24), DA + A + SC (*n* = 22), and DA + A only (*n* = 22). Most participants (89%) heard about the study via university channels (e.g., student mailing lists, campaigns webpage, campus posters), while 11% cited word of mouth or posters in local cafés. As the target sample size was reached early, it was not necessary to expand recruitment beyond these initial strategies.

##### 5.2.1.2 Retention

Fifty‐three participants (58%) completed the postintervention questionnaire, and 54 (59%) completed the follow‐up. Retention rates were similar across intervention components at both time points, ranging from 55% (24/44) to 63% (30/48).

##### 5.2.1.3 Data Analysis

All outcome and mechanism data were successfully collected, allowing exploration of the feasibility of the planned analyses (Box 1). Changes from baseline to postintervention and follow‐up across all conditions are presented in Table 5, indicating sensitivity to change across measures. Table 6 presents component‐level comparisons of postintervention outcomes (ER vs. no ER; SC vs. no SC), providing preliminary estimates of each component's effects on risk factors (Objective 1) and mechanisms (Objective 2). Table 7 presents correlations between change scores in mechanisms and outcomes, supporting the feasibility of examining associations between improvements in mechanisms and reductions in risk factors (Objective 3a). Analyses for Objective 3b (i.e., whether baseline levels of mechanisms or risk factors moderate component effectiveness) were not conducted due to limited sample size but are considered feasible for the main trial, given that moderator data were complete and reliable. Follow‐up data were not analyzed for Objectives 1–3 but are considered feasible to include, given acceptable retention and complete data among follow‐up completers.

**TABLE 5:** Changes in outcomes and targeted mechanisms from baseline to postintervention and follow‐up, pooled across all conditions.

Measure	Pre–post (*n* = 53)				Pre–FU (*n* = 54)			
	Pre *M* (SD)	Post *M* (SD)	*M*_diff_ (SD)	Cohen's *d* [95% CI]	Pre *M* (SD)	FU *M* (SD)	*M*_diff_ (SD)	Cohen's *d* [95% CI]
Weight and shape concerns	3.05 (1.41)	2.09 (1.42)	−0.95 (1.06)	0.90 [0.58–1.22]	3.13 (1.44)	2.38 (1.53)	−0.75 (1.20)	0.63 [0.33–0.92]
Negative affect	22.11 (7.34)	19.70 (7.71)	−2.42 (5.36)	0.45 [0.17–0.73]	22.33 (7.24)	20.76 (8.10)	−1.57 (6.68)	0.24 [−0.04–0.51]
Mindfulness	46.06 (7.33)	50.00 (8.01)	3.94 (7.43)	0.53 [0.24–0.82]	45.69 (6.97)	48.44 (7.45)	2.75 (6.94)	0.40 [0.12–0.67]
Emotion regulation difficulties	2.63 (0.78)	2.29 (0.74)	−0.34 (0.57)	0.60 [0.31–0.89]	2.66 (0.76)	2.36 (0.72)	−0.30 (0.54)	0.55 [0.26–0.84]
Self‐compassion	2.78 (0.60)	3.15 (0.70)	0.38 (0.54)	0.70 [0.40–1.00]	2.76 (0.59)	3.04 (0.63)	0.28 (0.57)	0.49 [0.20–0.77]

*Note*: Cohen's *d* was calculated as the mean difference divided by the standard deviation of the difference scores.

Abbreviations: FU = 1‐month follow‐up; *M* = mean; *M*_diff_ = mean difference (post − pre or follow‐up − pre); Post = postintervention (3 weeks); Pre = baseline; SD = standard deviation.

**TABLE 6:** Differences in postintervention scores between participants who received vs. did not receive each component, controlling for baseline.

Measure	ER+ (*n* = 29) Adj. *M* (SE)	ER– (*n* = 24) Adj. *M* (SE)	*M*_diff_ [95% CI]	*η* ^2^ *ₚ*	SC+ (*n* = 27) Adj. *M* (SE)	SC– (*n* = 26) Adj. *M* (SE)	*M*_diff_ [95% CI]	*η* ^2^ *ₚ*
Weight and shape concerns	2.34 (0.18)	1.79 (0.20)	0.55 [0.00, 1.09]	0.08	2.27 (0.20)	1.91 (0.20)	0.36 [−0.21, 0.93]	0.03
Negative affect	20.33 (0.96)	18.94 (1.06)	1.39 [−1.49, 4.27]	0.02	18.95 (0.99)	20.47 (1.01)	−1.52 [−4.37, 1.33]	0.02
Mindfulness	48.65 (1.26)	51.64 (1.39)	−2.99 [−6.80, 0.82]	0.05	50.64 (1.33)	49.33 (1.35)	1.31 [−2.50, 5.13]	0.01
Emotion regulation difficulties	2.24 (0.10)	2.34 (0.11)	−0.10 [−0.39, 0.19]	0.01	2.26 (0.10)	2.32 (0.10)	−0.06 [−0.35, 0.23]	< 0.01
Self‐compassion	3.17 (0.10)	3.14 (0.11)	0.03 [−0.26, 0.33]	< 0.01	3.29 (0.10)	3.01 (0.10)	0.28 [0.00, 0.56]	0.07

Abbreviations: Adj. *M* = estimated marginal mean (controlling for baseline); ER–/SC– = did not receive the respective component; ER+/SC+ = received the emotion regulation/self‐compassion component; *M*_diff_ = mean difference in the direction of component – no component, with 95% confidence interval; SE = standard error; *η* ^2^_p_ = partial eta squared.

**TABLE 7:** Correlations among change scores in outcomes and targeted mechanisms, pooled across conditions.

	Δ Outcome	
Δ Mechanism	Weight and shape concerns	Negative affect
Mindfulness	−0.12	−0.32
Emotion regulation difficulties	0.33	0.34
Self‐compassion	−0.13	−0.36

*Note*: Values are Pearson correlation coefficients between pre to post change scores. Negative values indicate that increases in mechanisms are associated with decreases in outcomes.

#### 5.2.2 Feasibility of the Intervention Design

##### 5.2.2.1 Engagement

Across the full sample (*N* = 92), participants completed a mean of 4.0 (SD = 3.8) of the 9 modules (44.2%). Completion patterns were bimodal: 55% completed two or fewer modules, while 34% completed eight or more (Figure 1). Together, these engagement patterns accounted for 89% of the sample, indicating a tendency toward essential or near‐complete engagement. Overall, 80% of participants (74/92) met the threshold for essential use by completing at least one module.

**FIGURE 1:**
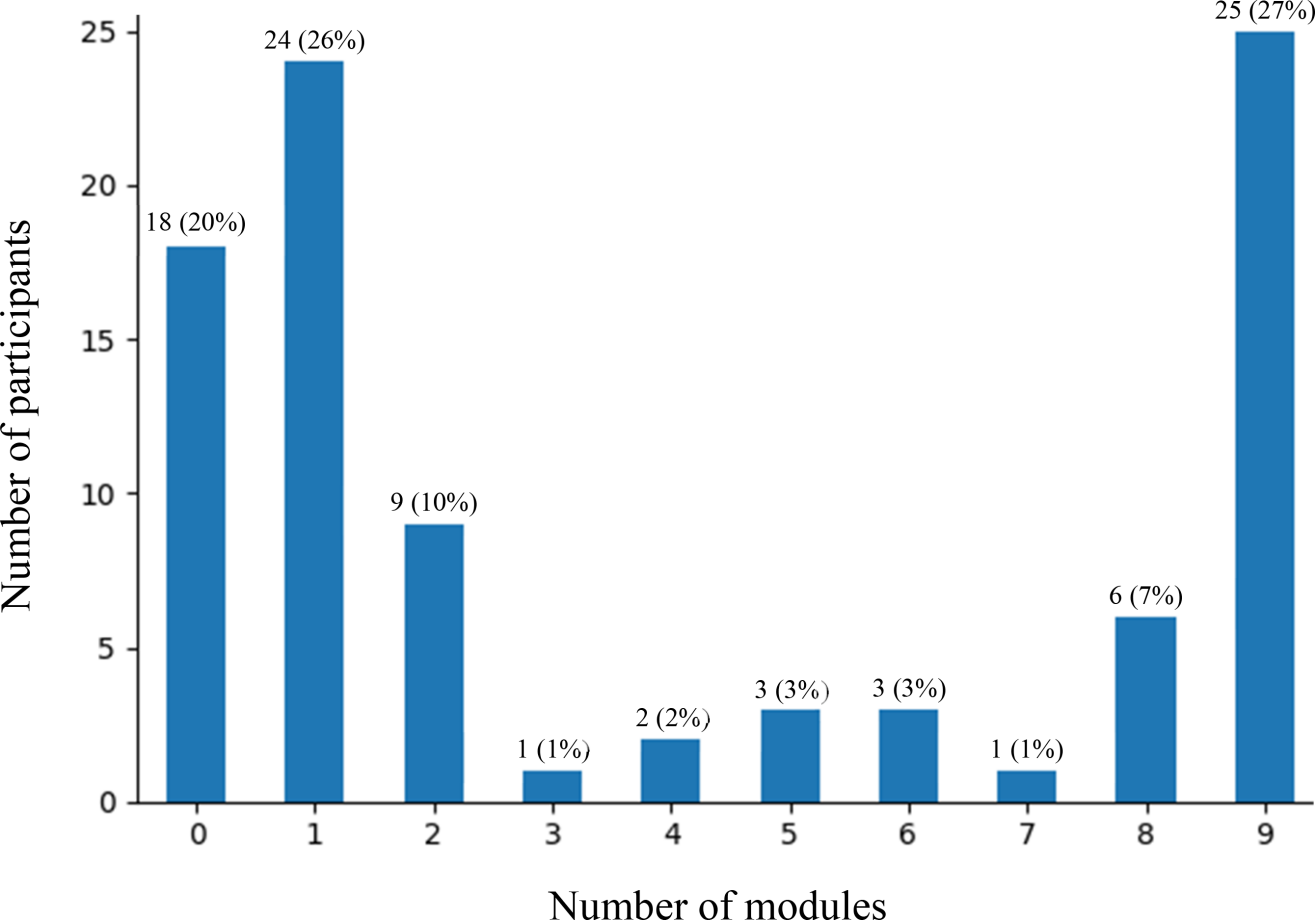
Number of participants completing each total number of modules (all conditions combined).

All participants accessed at least some intervention content, defined as the introduction and/or a module. Most (82/92; 89%) opened Module 1. Among all participants, total time spent engaging with intervention content ranged from 0.5 to 108.2 min (*M* = 11.9, SD = 16.4), and time spent specifically on modules ranged from 0 to 104.8 min (*M* = 9.6, SD = 15.6). Completion rates declined between Modules 1 and 3 but remained relatively stable from Modules 4 to 9, suggesting a steep initial drop‐off followed by consistent engagement among continuing users (see Supporting Information Data S4 for module‐by‐module completion rates).

Engagement did not differ by component. Average module completion was similar for participants who received ER (*M* = 3.9, SD = 3.9) versus those who did not (*M* = 4.1, SD = 3.6), and for those who received SC (*M* = 4.1, SD = 3.8) versus those who did not (*M* = 3.8, SD = 3.7). Module completion patterns were also comparable across components (see Supporting Information Data S4). The proportion of participants meeting the essential use threshold was 77% (37/48) for those who received ER versus 84% (37/44) who did not, and 78% (36/46) for those who received SC versus 83% (38/46) who did not. Time spent engaging with the intervention was highly variable but showed similar distributions across components: ER received (*M* = 11.4, SD = 14.07) versus not received (*M* = 12.6, SD = 18.8); SC received (*M* = 12.6, SD = 15.5) versus not received (*M* = 11.2, SD = 17.4).

##### 5.2.2.2 Acceptability

Participants (*N* = 92) generally reported positive experiences with *eMindfulness Mode* (see Table 8; full response distributions are in Supporting Information Data S4). Most liked the intervention (85%), found it required little effort to use (70%), and considered it fair for people with body image concerns (85%). Over half (55%) perceived improvements in their body image. Additionally, 89% understood how the program was intended to work conceptually, 79% felt confident using it, and only 21% felt it interfered with other priorities. On a 5‐point scale (1 = *completely unacceptable*, 5 = *completely acceptable*), the mean acceptability score was 4.28 (SD = 0.74), and no participants rated the intervention as unacceptable. Acceptability ratings were similar regardless of ER or SC inclusion; for example, general acceptability scores ranged from *M* = 4.25–4.31 across components, with similar patterns observed for other TFA constructs.

**TABLE 8:** TFA‐based acceptability scores for the full sample and by intervention components.

TFA construct and item	Full sample (*n* = 53)	ER+ (*n* = 29)	ER– (*n* = 24)	SC+ (*n* = 27)	SC– (*n* = 26)
Affective attitude: Did you like or dislike *eMindfulness Mode*?	3.94 (0.69)	3.90 (0.72)	4.00 (0.66)	3.96 (0.65)	3.92 (0.74)
Burden: How much effort did it take to use *eMindfulness Mode*?	2.53 (0.95)	2.38 (0.90)	2.71 (1.00)	2.67 (1.04)	2.38 (0.85)
Ethicality: How fair *is eMindfulness Mode* for people with body image concerns?	4.02 (0.57)	4.00 (0.53)	4.04 (0.62)	3.89 (0.51)	4.15 (0.61)
Perceived effectiveness: *eMindfulness Mode* has improved my body image	3.45 (0.77)	3.48 (0.87)	3.42 (0.65)	3.41 (0.80)	3.50 (0.76)
Intervention coherence: It is clear to me how *eMindfulness Mode* could help improve body image	4.11 (0.64)	4.10 (0.62)	4.13 (0.68)	4.07 (0.55)	4.15 (0.73)
Self‐efficacy: How confident did you feel about using *eMindfulness Mode*?	3.89 (0.89)	3.86 (0.83)	3.92 (0.97)	3.89 (0.93)	3.88 (0.86)
Opportunity costs: Using *eMindfulness Mode* interfered with my other priorities	2.40 (1.10)	2.24 (1.02)	2.58 (1.18)	2.33 (1.07)	2.46 (1.14)
General acceptability: How acceptable was *eMindfulness Mode* to you?	4.28 (0.74)	4.31 (0.71)	4.25 (0.79)	4.30 (0.78)	4.27 (0.72)

*Note*: Values represent *M* (SD) on a 5‐point scale. Anchors varied by item. Response options ranged from 1 = *Strongly dislike*, *No effort at all*, *Very unfair*, *Strongly disagree*, *Very unconfident*, *Completely unacceptable* to 5 = *Strongly like*, *Huge effort*, *Very fair*, *Strongly agree*, *Very confident*, *Completely acceptable*.

Abbreviations: ER–/SC– = did not receive the emotion regulation/self‐compassion component; ER+/SC+ = received the emotion regulation/self‐compassion component; TFA = theoretical framework of acceptability.

Of the 51 participants who provided feedback, the most frequently mentioned aspect was communication. Nearly half (23/51) found the email reminders helpful for staying on track, and six suggested sending them more frequently. Twelve participants commented on content format and presentation. Some found the program too text‐heavy (4/51), while others appreciated the audio and interactive elements (3/51). Several suggested incorporating more images and varied formats (5/51). Six participants mentioned usability, including suggestions for clearer navigation (3/51) and easier access to notes, exercises, and videos (2/51).

## 6 Discussion

### 6.1 Principal Findings

This study evaluated the feasibility of conducting a factorial ORCT in preparation for a full‐scale trial testing the effectiveness of adding ER and SC to a digital MBI targeting risk factors for disordered eating. In line with the UK Medical Research Council framework (Skivington et al. 2021), we examined key uncertainties in recruitment, retention, data analysis, and intervention engagement and acceptability. Findings support progression to a main trial and provide a crucial foundation for improving the effectiveness and engagement of digital MBIs.

Recruitment was highly successful: we exceeded our target sample size of 80 within the first 2 weeks. Our strategies attracted participants with elevated levels on key risk factors, suggesting the study reached individuals at high risk or in transition to an eating disorder (Levine 2019)—a relevant audience for the intervention.

Retention rates were 58% at postintervention and 59% at follow‐up, exceeding the minimum progression criterion though falling short of the prespecified green threshold. While not ideal, these rates are within the range typically reported for similar digital interventions and may be considered reasonable under pragmatic, real‐world conditions, especially given the absence of intensive retention strategies (e.g., researcher contact). Notably, they exceed those reported in a trial of digital mindfulness and gratitude micro‐interventions for body satisfaction, where only 27% of the intervention group completed the postassessment after 21 days (Fuller‐Tyszkiewicz et al. 2019), and align with rates observed in other online disordered eating prevention trials (Wilksch et al. 2018, 2025).

Although strategies such as requiring researcher contact at enrolment or offering monetary incentives seem to improve retention, they are likely to reduce ecological validity by introducing procedures not typical of real‐world app use (Linardon and Fuller‐Tyszkiewicz 2020). No consistent participant‐level predictors of attrition in e‐mental health studies have been identified (Linardon and Fuller‐Tyszkiewicz 2020), yet this remains an important direction for future research, as such insights could support more targeted retention strategies (e.g., identifying subgroups who might benefit from additional support).

All planned analyses were feasible, and all measures demonstrated sensitivity to change, supporting the viability of evaluating the main trial objectives. Exploratory ANCOVAs suggested no reliable differences in outcomes between components, though there were weak indications of benefit for self‐compassion with SC and for weight and shape concerns without ER. Overall, the evaluation design appears appropriate for a larger trial.

Engagement met the progression criterion: 80% of participants completed Module 1, well above the 50% threshold and rates reported in similar trials (33%–50%; Wilksch et al. 2018, 2025). Most dropouts occurred after Module 1, supporting the rationale for delivering core content early so participants are exposed to key concepts even if they disengage. Engagement patterns were consistent across components. Among those continuing, 35%–41% completed Modules 3–9, again exceeding comparable rates (Wilksch et al. 2018, 2025). The average completion rate (44%) was slightly higher than the 40% reported in digital well‐being interventions (Groot et al. 2023). All participants accessed intervention content, surpassing the 29% nonengagement rate reported in a meta‐analysis of digital eating disorder prevention programs (Linardon et al. 2020).

Acceptability was high and similar across components, with no participants rating the intervention as unacceptable and a mean acceptability rating of 4.28 out of 5. This compares favorably to similar microinterventions, where engagement ratings averaged slightly below the neutral midpoint (*M* = 2.89; Fuller‐Tyszkiewicz et al. 2019), and underscores the value of user‐centered development approaches, such as the PBA used here (Yardley et al. 2015). Feedback valued the reminders and identified areas for improvement in content format and usability. This aligns with existing evidence on the importance of communication in digital MBIs (Osborne et al. 2023) and multimedia content in eating disorder prevention programs (Kristoffersen et al. 2022). Future iterations should retain these supportive features while enhancing navigation and visual design.

### 6.2 Strengths and Limitations

This study had several methodological strengths, including preregistered progression criteria, the use of a formal framework for sample size estimation focused on process outcomes, and an innovative factorial ORCT design that enabled evaluation of individual components. It also included a comprehensive assessment of the intervention and evaluation design, aligning with MRC guidance, which emphasizes feasibility testing as a critical step in determining whether to proceed to full‐scale evaluation (Skivington et al. 2021).

However, there are some limitations. Most participants were women, highly educated, and from low‐deprivation areas, which limits the generalizability of the findings. Future research should incorporate more targeted recruitment strategies and greater involvement of underrepresented groups. We also did not recruit any participants aged 16–17, due to the success of initial recruitment in university and community settings. Given that adolescence is a critical period for the development of disordered eating, future trials should prioritize school‐based recruitment to better reach this age group. Retention fell below the green threshold, and there was substantial drop‐off after the first few modules, highlighting the need to explore how retention might be improved and which patterns of engagement are most predictive of benefit, reflecting a shift toward “effective engagement” as a more meaningful metric (Yardley et al. 2016).

### 6.3 Future Research

Findings support progression to a fully powered factorial trial. Despite lower sustained engagement, the large pre–post and body image reduction in weight and shape concerns suggests promise even with brief use of this low‐cost, autonomous digital intervention. Future work should explore scalable strategies to enhance retention, such as codeveloping the content and format of automated reminders with target users or personalizing reminders based on individual progress. Such low‐burden enhancements may improve retention without compromising ecological validity. For example, personalized messages developed with patients have shown promise in supporting outcomes over 12 months postintervention (Malins et al. 2020), highlighting their potential as a low‐cost, scalable addition to digital interventions, while their specific impact on trial retention warrants investigation.

Further development should incorporate structured elements of the PBA to systematically address participant feedback and ensure the intervention remains motivating and persuasive (Yardley et al. 2015). Coproducing recruitment and intervention materials with underrepresented groups (e.g., men, younger adolescents) will also help extend reach and relevance. Ultimately, the full trial will determine which components warrant inclusion in a streamlined, scalable intervention (Collins et al. 2024).

### 6.4 Implications for Interventions

This research lays the foundation for developing more efficient digital MBIs by combining components that offer the greatest benefit with the least user burden. Such interventions are well suited to address the growing need for accessible, scalable prevention and early intervention for disordered eating and body image concerns in young people (Beat 2022)—a recognized global health priority (Rodgers et al. 2023). Optimized programs could be delivered in educational, healthcare, and community settings to support early intervention at scale.

## 7 Conclusion

This feasibility study supports progression to a full‐scale factorial ORCT to evaluate ER and SC as additional components in a digital MBI to reduce risk factors for disordered eating. The trial and intervention designs were acceptable and implementable, providing a strong foundation for developing an effective, engaging, and scalable intervention.

## Author Contributions

**Emma L. Osborne:** conceptualization, methodology, writing – original draft, writing – review and editing, formal analysis, funding acquisition, investigation, project administration. **Ben Ainsworth:** conceptualization, methodology, writing – review and editing, supervision. **Nic Hooper:** conceptualization, methodology, writing – review and editing, supervision. **Paul Chadwick:** conceptualization, methodology, writing – review and editing, supervision. **Melissa J. Atkinson:** conceptualization, methodology, writing – review and editing, supervision, funding acquisition.

## Conflicts of Interest

The authors declare no conflicts of interest.

## Supporting information


**Data S1:** Components of mindfulness‐based interventions.

**Data S2:** TIDieR checklist.

**Data S3:** Overview of modules and intervention screenshots.

**Data S4:** Results.

## Data Availability

The data that support the findings of this study are available from the corresponding author upon reasonable request.
