# Supplementary material for: Optimizing a Digital Mindfulness‐Based Intervention to Reduce Risk Factors for Disordered Eating: A Multiphase Optimization Strategy (MOST) Feasibility Trial
Source: Int J Eat Disord. 2025 Sep 16;58(12):2331–43. doi: 10.1002/eat.24546 (PMC13766239; doi:10.1002/eat.24546)
Supplement: Supplementary file 1 [file EAT-58-2331-s001.docx]

**Article title**: Optimising a Digital Mindfulness-Based Intervention to Reduce Risk Factors for Disordered Eating: A Multiphase Optimisation Strategy (MOST) Feasibility Trial

**Journal name**: *International Journal of Eating Disorders (IJED)*

**Author names**: Emma L. Osborne, Ben Ainsworth, Nic Hooper, Paul Chadwick, Melissa J. Atkinson

**Corresponding author**: Emma L. Osborne, Department of Psychology, University of Bath, Claverton Down, Bath, BA2 7AY, UK; Email: elo25@bath.ac.uk

# Supplementary File 3: Overview of Modules and Intervention Screenshots

**Table 1**

Overview of module themes, topics, and activities across intervention conditions

| Module | Themes, topics, and activities | DA+A | DA+A + ER | DA+A + SC | DA+A + ER + SC |
| --- | --- | --- | --- | --- | --- |
| Introduction | **Topics** | Overview and goals; what to expect; making the most of your journey. | Overview and goals; what to expect; making the most of your journey. | Overview and goals; what to expect; making the most of your journey. | Overview and goals; what to expect; making the most of your journey. |
| 1 | **Theme** | A new way of coping. | **An alternative** way of coping. | A new way of coping. | **An alternative** way of coping. |
|  | **Topics** | Psychoeducation on body image; mindfulness as a way of coping with negative body-related experiences; paying attention in the present. | Psychoeducation on body image; **common responses to intense emotions and body-related distress (e.g., avoidance, rumination) and their costs**; mindfulness as a **helpful alternative coping strategy for these emotions**; paying attention in the present. | Psychoeducation on body image; **recognising body-related self-criticism and understanding its downsides**; mindfulness as a **foundation for developing a more accepting attitude and responding to such self-criticism with compassion and kindness**; paying attention in the present. | Psychoeducation on body image; **common responses to intense emotions and body-related distress (e.g., avoidance, rumination) and their costs**; mindfulness as a **helpful alternative coping strategy for these emotions**; **recognising body-related self-criticism and understanding its downsides**; mindfulness as a **foundation for developing a more accepting attitude and responding to such self-criticism with compassion and kindness**; paying attention in the present. |
|  | **Activities** | Raisin exercise and reflection. | **Brainstorm personal coping strategies and reflect on their unintentional downsides**; **thought suppression exercise**; **thought magnification exercise**; raisin exercise and reflection. | **“Which coach?” scenario to demonstrate the impact of self-criticism vs. self-compassion**; **notice self-critical thoughts and reflect on their impact**; raisin exercise and reflection. | **Brainstorm personal coping strategies and reflect on their unintentional downsides**; **thought suppression exercise**; **thought magnification exercise**; **“Which coach?” scenario to demonstrate the impact of self-criticism vs. self-compassion**; **notice self-critical thoughts and reflect on their impact**; raisin exercise and reflection. |
| 2 | **Theme** | Getting started with mindfulness. | Getting started with mindfulness. | Getting started with mindfulness. | Getting started with mindfulness. |
|  | **Topics** | What mindfulness is and how it can help; key attitudes of mindfulness; beginning your mindfulness practice. | What mindfulness is and how it can help **(e.g., manage emotions in the long run)**; key attitudes of mindfulness; beginning your mindfulness practice. | What mindfulness is and how it can help **(e.g., treat ourselves with more kindness and compassion)**; key attitudes of mindfulness; beginning your mindfulness practice. | What mindfulness is and how it can help **(e.g., manage emotions and treat ourselves with more kindness and compassion)**; key attitudes of mindfulness; beginning your mindfulness practice. |
|  | **Activities** | Mindfulness of breath and body, with reflection. | Mindfulness of breath and body, with reflection **(e.g., how it might help when experiencing intense emotions)**. | Mindfulness of breath and body, with reflection **(e.g., how it might help when experiencing self-critical thoughts)**. | Mindfulness of breath and body, with reflection **(e.g., how it might help when experiencing self-critical thoughts or intense emotions)**. |
| 3 | **Theme** | Being present and aware. | Being present and aware. | Being present and aware. | Being present and aware. |
|  | **Topics** | Becoming aware; brief mindfulness throughout the day; making mindfulness work for you; being present. | Becoming aware; brief mindfulness throughout the day; making mindfulness work for you; being present. | Becoming aware; brief mindfulness throughout the day; making mindfulness work for you; being present. | Becoming aware; brief mindfulness throughout the day; making mindfulness work for you; being present. |
|  | **Activities** | 3-minute breathing space (scheduled) with reflection; bringing awareness to daily activities. | 3-minute breathing space (scheduled), with reflection **on how it might be helpful in situations or difficult emotions that trigger unhelpful coping strategies**; bringing awareness to daily activities. | 3-minute breathing space (scheduled), with reflection **on how it might be helpful in situations that trigger your self-critical voice**; bringing awareness to daily activities. | 3-minute breathing space (scheduled), with reflection **on how it might be helpful in situations that trigger your self-critical voice or involve difficult emotions and unhelpful coping**; bringing awareness to daily activities. |
| 4 | **Theme** | Overcoming mindfulness challenges. | Overcoming mindfulness challenges. | Overcoming mindfulness challenges. | Overcoming mindfulness challenges. |
|  | **Topics** | Common challenges in practising mindfulness; personal barriers to practice; tips for dealing with barriers. | Common challenges in practising mindfulness; personal barriers to practice **(e.g., when using mindfulness instead of usual coping strategies)**; tips for dealing with barriers. | Common challenges in practising mindfulness; personal barriers to practice **(e.g., during negative self-talk about your body)**; tips for dealing with barriers. | Common challenges in practising mindfulness; personal barriers to practice **(e.g., when using mindfulness instead of usual coping strategies or during negative self-talk about your body)**; tips for dealing with barriers. |
|  | **Activities** | Practise mindfulness during a positive body-related activity; reflect on challenges with learning mindfulness; identify tips for overcoming these challenges. | Practise mindfulness during a positive body-related activity; reflect on challenges with learning mindfulness **and using it to cope with negative feelings about your body**; identify tips for overcoming these challenges. | Practise mindfulness during a positive body-related activity; reflect on challenges with learning mindfulness **and being kind to yourself**; identify tips for overcoming these challenges. | Practise mindfulness during a positive body-related activity; reflect on challenges with learning mindfulness**, using it to cope with negative feelings about your body, and being kind to yourself**; identify tips for overcoming these challenges. |
| 5 | **Theme** | Relating differently to experiences. | Relating differently to experiences. | Relating differently to experiences. | Relating differently to experiences. |
|  | **Topics** | Taking a step back from the mind; the importance of how we relate to our experience. | Taking a step back from the mind **(e.g., stepping back from intense emotions that may lead to unhelpful behaviours)**; the importance of how we relate to our experience. | Taking a step back from the mind **(e.g., stepping back from self-critical thoughts that may lead to unhelpful behaviours)**; the importance of how we relate to our experience. | Taking a step back from the mind **(e.g., stepping back from intense emotions or self-critical thoughts that may lead to unhelpful behaviours)**; the importance of how we relate to our experience. |
|  | **Activities** | Distancing thought exercise (“I am having the thought that…”), applied to any automatic negative thoughts. | Distancing thought exercise (“I am having the thought that…”), **applied to intense emotions.** | Distancing thought exercise (“I am having the thought that…”), **applied to self-criticism.** | Distancing thought exercise (“I am having the thought that…”), **applied to intense emotions, self-criticism, and automatic negative thoughts.** |
| 6 | **Theme** | We are not our experience. | We are not our experience. | We are not our experience. | We are not our experience. |
|  | **Topics** | Mindfulness and the media; separating ourselves from our experience. | Mindfulness and the media; separating ourselves from our experience. | Mindfulness and the media; separating ourselves from our experience. | Mindfulness and the media; separating ourselves from our experience. |
|  | **Activities** | *Mind as a cinema screen* exercise (generic) and reflection; mindfulness of a body-related experience, including physical discomfort (e.g., experiencing tension or discomfort in your body), with reflection. | *Mind as a cinema screen* exercise **(tailored to difficult emotions)** and reflection; mindfulness of a body-related experience, **including difficult emotions (e.g., feeling overwhelmed by negative emotions about your body)**, with reflection. | *Mind as a cinema screen* exercise **(tailored to self-critical thoughts)** and reflection; mindfulness of a body-related experience, **including self-critical experiences (e.g., criticising your body)**, with reflection. | *Mind as a cinema screen* exercise **(tailored to difficult emotions and self-critical thoughts)** and reflection; mindfulness of a body-related experience, **including difficult moments (e.g., feeling overwhelmed by negative emotions or criticising your body)**, with reflection. |
| 7 | **Theme** | Practising non-judgement. | Practising non-judgement. | Practising non-judgement. | Practising non-judgement. |
|  | **Topics** | Understanding and releasing judgement; impact of judgement; responding to judgement; welcoming all experiences; experiences as visitors. | Understanding and releasing judgement; impact of judgement; responding to judgement; welcoming all experiences; experiences as visitors. | Judgement and self-criticism; impact of judgement **and self-criticism**; responding to judgement **and self-criticism**; welcoming all experiences; experiences as visitors. | Judgement and self-criticism; impact of judgement **and self-criticism**; responding to judgement **and self-criticism**; welcoming all experiences; experiences as visitors. |
|  | **Activities** | Reflect on the impact of being judgemental toward experiences; non-judgemental response exercise applied to negative body-related experiences; read and reflect on *The Guest House* poem. | Reflect on the impact of being judgemental toward experiences; non-judgemental response exercise applied to **negative emotions about your body**; read and reflect on *The Guest House* poem. | Reflect on the impact of being critical toward experiences; non-judgemental response exercise applied to **self-critical thoughts about your body**; read and reflect on *The Guest House* poem. | Reflect on the impact of being critical toward experiences; non-judgemental response exercise applied to **negative emotions and self-critical thoughts about your body**; read and reflect on *The Guest House* poem. |
| 8 | **Theme** | Strengthening your mindfulness muscle. | Strengthening your mindfulness muscle. | **Treating yourself with kindness.** | **Treating yourself with kindness.** |
|  | **Topics** | Non-judgement towards experience; strengthening your mindfulness muscle. | Non-judgement towards experience; strengthening your mindfulness muscle. | **Self-criticism and self-compassion**; **how self-compassion can help**; **practising self-compassion**; strengthening your mindfulness muscle. | **Self-criticism and self-compassion**; **how self-compassion can help**; **practising self-compassion**; strengthening your mindfulness muscle. |
|  | **Activities** | Respond mindfully to media and reflection; mindful mirror exercise (responding to negative body image experiences with non-judgment); 3-minute breathing space as a tool for coping with negative experiences. | Respond mindfully to media and reflect; mindful mirror exercise **(responding to intense emotions or distress related to your body with non-judgement)**; 3-minute breathing space as a tool for coping with **emotional distress**. | **Brainstorm the costs of self-criticism**; **self-compassionate response activity**; respond mindfully to media and reflection; mindful mirror exercise **(responding to self-critical thoughts with self-compassion)**; 3-minute breathing space as a tool for coping with **self-criticism**. | **Brainstorm the costs of self-criticism**; **self-compassionate response activity**; respond mindfully to media and reflect; mindful mirror exercise **(responding to intense emotions and self-critical thoughts related to your body with non-judgement and self-compassion)**; 3-minute breathing space as tool for coping with **self-criticism and emotional distress**. |
| 9 | **Theme** | Beyond *eMindfulness Mode*. | Beyond *eMindfulness Mode*. | Beyond *eMindfulness Mode*. | Beyond *eMindfulness Mode*. |
|  | **Topics** | Reflecting on your journey; preparing for future pressures; finishing *eMindfulness Mode*; accessing additional support. | Reflecting on your journey; preparing for future pressures; finishing *eMindfulness Mode*; accessing additional support. | Reflecting on your journey; preparing for future pressures; finishing *eMindfulness Mode*; accessing additional support. | Reflecting on your journey; preparing for future pressures; finishing *eMindfulness Mode*; accessing additional support. |
|  | **Activities** | Mindfulness of the body; making a commitment; developing a personal action plan with example situations related to negative body image; creating personal mindfulness statements with general examples. | Mindfulness of the body; making a commitment; developing a personal action plan with example situations **tailored to times when you might experience difficult emotions**; creating personal mindfulness statements with **examples related to emotions**. | Mindfulness of the body; making a commitment; developing a personal action plan with example situations **tailored to times when you might be self-critical**; creating personal mindfulness statements with **examples involving self-compassion**. | Mindfulness of the body; making a commitment; developing a personal action plan with example situations **tailored to times when you might experience difficult emotions or be self-critical**; creating personal mindfulness statements with examples **related to emotions and self-compassion**. |
| *Note.* DA+A = decentred awareness and acceptance of present-moment experience; ER = emotion regulation; SC = self-compassion. Bolded text indicates how ER and SC components were embedded through examples, exercises, and reflections by adjusting the framing, clarifying the contexts in which to apply these skills, and increasing emphasis to support the learning of DA+A. DA+A content was held constant across conditions. | | | | | |

**Figure 1.** Example screenshots from the intervention.
