# Supplementary material for: Optimizing a Digital Mindfulness‐Based Intervention to Reduce Risk Factors for Disordered Eating: A Multiphase Optimization Strategy (MOST) Feasibility Trial
Source: Int J Eat Disord. 2025 Sep 16;58(12):2331–43. doi: 10.1002/eat.24546 (PMC13766239; doi:10.1002/eat.24546)
Supplement: Supplementary file 3 [file EAT-58-2331-s003.docx]

**Article title**: Optimising a Digital Mindfulness-Based Intervention to Reduce Risk Factors for Disordered Eating: A Multiphase Optimisation Strategy (MOST) Feasibility Trial

**Journal name**: *International Journal of Eating Disorders (IJED)*

**Author names**: Emma L. Osborne, Ben Ainsworth, Nic Hooper, Paul Chadwick, Melissa J. Atkinson

**Corresponding author**: Emma L. Osborne, Department of Psychology, University of Bath, Claverton Down, Bath, BA2 7AY, UK; Email: elo25@bath.ac.uk

# Supplementary File 1: Components of Mindfulness-Based Interventions

**Table S1.** Examples of components in mindfulness-based interventions.

| Component | Description of component (examples from MBIs listed below) | | | | | | | | |
| --- | --- | --- | --- | --- | --- | --- | --- | --- | --- |
| DA+A | Decentred awareness and acceptance of present-moment experience. | | | | | | | | |
| Stress management | Psychophysiology of stress and emotions, applying mindfulness in stressful situations, and coping with distressing situations and life stressors. | | | | | | | | |
| Cognitive restructuring | Identifying and letting go of ineffective patterns in thinking. | | | | | | | | |
| Values | Clarifying personal values, emphasising the importance of living in accordance with these core values, and committing to act in line with them. | | | | | | | | |
| Emotion regulation | Understanding emotions and the various ways to manage them, including the costs of unhelpful strategies, and reducing negative emotional states. | | | | | | | | |
| Distress tolerance | Managing emotional distress arising from aversive life events, crises. | | | | | | | | |
| Self-compassion | Developing a kind, compassionate, and accepting attitude toward oneself, and cultivating a compassionate inner voice. | | | | | | | | |
| Interpersonal skills | Interacting effectively with others, managing challenging relationships, and pursuing personal goals while maintaining social connections. | | | | | | | | |
| Appreciation | Cultivating gratitude and relating to the valued aspects of oneself and one’s life with appreciation. | | | | | | | | |
| MBI | DA+A | Stress management | Cognitive restructuring | Values | Emotion regulation | Distress tolerance | Self-compassion | Interpersonal skills | Appreciation |
| MBSR | X | X |  |  |  |  |  |  |  |
| MBCT | X | X | X |  | X | X |  |  |  |
| ACT | X |  |  | X |  | X |  |  |  |
| DBT | X |  |  |  | X | X |  |  |  |
| MB-EAT | X |  |  |  | X |  | X |  |  |
| MSC | X |  |  | X | X |  | X | X | X |
| TMM | X |  |  |  | X |  | X |  |  |
| MBC | X | X |  |  | X |  |  | X |  |
| *Note*. DA+A = decentred awareness and acceptance of present-moment experience (Bishop et al., 2004; Lindsay & Creswell, 2017; Shapiro et al., 2005); MBI = mindfulness-based intervention; MBSR = Mindfulness-Based Stress Reduction (Kabat-Zinn, 1982, 1990); MBCT = Mindfulness-Based Cognitive Therapy (Segal et al., 2002); ACT = Acceptance and Commitment Therapy (Hayes et al., 1999); DBT = Dialectical Behaviour Therapy (Linehan, 1993); MB-EAT = Mindfulness-Based Eating Awareness Training (Kristeller & Wolever, 2010); MSC = Mindful Self-Compassion (Neff & Germer, 2013); TMM = The Mindfulness Mode (Atkinson & Wade, 2015, 2016); MBC = Mindfulness-Based Coping (Tharaldsen & Bru, 2012). | | | | | | | | | |

**References**

Atkinson, M. J., & Wade, T. D. (2015). Mindfulness-based prevention for eating disorders: A school-based cluster randomized controlled study. *International Journal of Eating Disorders*, *48*(7), 1024–1037. https://doi.org/10.1002/eat.22416

Atkinson, M. J., & Wade, T. D. (2016). Does mindfulness have potential in eating disorders prevention? A preliminary controlled trial with young adult women. *Early Intervention in Psychiatry*, *10*(3), 234–245. https://doi.org/10.1111/eip.12160

Hayes, S. C., Strosahl, K., & Wilson, K. G. (1999). *Acceptance and commitment therapy: An experiential approach to behavior change*. Guilford Press.

Kabat-Zinn, J. (1982). An outpatient program in behavioral medicine for chronic pain patients based on the practice of mindfulness meditation: Theoretical considerations and preliminary results. *General Hospital Psychiatry*, *4*(1), 33–47. https://doi.org/10.1016/0163-8343(82)90026-3

Kabat-Zinn, J. (1990). *Full catastrophe living: Using the wisdom of your mind and body to face stress, pain, and illness*. Delacorte.

Kristeller, J. L., & Wolever, R. Q. (2010). Mindfulness-based eating awareness training for treating binge eating disorder: The conceptual foundation. *Eating Disorders*, *19*(1), 49–61. https://doi.org/10.1080/10640266.2011.533605

Linehan, M. M. (1993). *Cognitive behavioral treatment of borderline personality disorder*. Guilford Press.

Neff, K. D., & Germer, C. K. (2013). A pilot study and randomized controlled trial of the mindful self-compassion program. *Journal of Clinical Psychology*, *69*(1), 28–44. https://doi.org/10.1002/jclp.21923

Segal, Z. V., Williams, J. M. G., & Teasdale, J. D. (2002). *Mindfulness-based cognitive therapy for depression: A new approach to preventing relapse*. Guilford Press.

Tharaldsen, K. B., & Bru, E. (2012). Evaluating the mindfulness-based coping program: An effectiveness study using a mixed model approach. *Mental Illness*, *4*(1), 43–51. https://doi.org/10.4081/mi.2012.e11
