# Supplementary material for: Optimizing a Digital Mindfulness‐Based Intervention to Reduce Risk Factors for Disordered Eating: A Multiphase Optimization Strategy (MOST) Feasibility Trial
Source: Int J Eat Disord. 2025 Sep 16;58(12):2331–43. doi: 10.1002/eat.24546 (PMC13766239; doi:10.1002/eat.24546)
Supplement: Supplementary file 4 [file EAT-58-2331-s004.docx]

**Article title**: Optimising a Digital Mindfulness-Based Intervention to Reduce Risk Factors for Disordered Eating: A Multiphase Optimisation Strategy (MOST) Feasibility Trial

**Journal name**: *International Journal of Eating Disorders (IJED)*

**Author names**: Emma L. Osborne, Ben Ainsworth, Nic Hooper, Paul Chadwick, Melissa J. Atkinson

**Corresponding author**: Emma L. Osborne, Department of Psychology, University of Bath, Claverton Down, Bath, BA2 7AY, UK; Email: elo25@bath.ac.uk

# Supplementary File 4: Results

## Demographics

| **Supplementary Table S1.** Demographic breakdown | | |
| --- | --- | --- |
| Category | *n* | % |
| Ethnicity | | |
| White | 48 | 52% |
| Asian or Asian British | 30 | 33% |
| Mixed or multiple ethnic groups | 8 | 9% |
| Black, Black British, Caribbean or African | 2 | 2% |
| Other ethnic group | 3 | 3% |
| Prefer not to say | 1 | 1% |
|  |  |  |
| Education Level | | |
| A Levels or equivalent | 54 | 59% |
| Undergraduate degree | 23 | 25% |
| Master's degree | 7 | 8% |
| No formal qualifications | 4 | 4% |
| Doctorate | 3 | 3% |
| Apprenticeship | 1 | 1% |
|  |  |  |
| Index of Multiple Deprivation (IMD) | | |
| Decile 1 (most deprived) | 1 | 1% |
| Decile 2 | 3 | 4% |
| Decile 3 | 10 | 13% |
| Decile 4 | 3 | 4% |
| Decile 5 | 4 | 5% |
| Decile 6 | 3 | 4% |
| Decile 7 | 2 | 3% |
| Decile 8 | 8 | 10% |
| Decile 9 | 5 | 6% |
| Decile 10 (least deprived) | 38 | 49% |

## Intervention engagement

Module #

Number of participants

**Figure S1** Number of participants who completed each intervention module (Modules 1–9).

74 (80%)

49 (53%)

37 (40%)

38 (41%)

36 (39%)

32 (35%)

34 (37%)

32 (35%)

34 (37%)

| **Figure S2** Number of participants completing each total number of modules (left panel) and number of participants who completed each intervention module (right panel). | |
| --- | --- |
| Received the emotion regulation component (*n* = 48) | |
| Number of modules  Number of participants  | Module #  Number of participants  |
| Did not receive the emotion regulation component (*n* = 44) | |
|   Number of modules  Number of participants |   Module #  Number of participants |

| **Figure S3** Number of participants completing each total number of modules (left panel) and number of participants who completed each intervention module (right panel). | |
| --- | --- |
| Received the self-compassion component (*n* = 46) | |
|   Number of modules  Number of participants |   Module #  Number of participants |
| Did not receive the self-compassion component (*n* = 46) | |
|   Number of modules  Number of participants |   Module #  Number of participants |

Intervention acceptability

| **Supplementary Table S2.** TFA-based acceptability responses for the full sample (*n* = 53) | | | |
| --- | --- | --- | --- |
| TFA construct and item | Response option | *n* | % |
| Affective attitude: Did you like or dislike *eMindfulness Mode*? | Strongly dislike | 0 | 0% |
|  | Dislike | 3 | 6% |
|  | No opinion | 5 | 9% |
|  | Like | 37 | 70% |
|  | Strongly like | 8 | 15% |
| Burden: How much effort did it take to use *eMindfulness Mode*? | No effort at all | 2 | 4% |
|  | A little effort | 35 | 66% |
|  | No opinion | 3 | 6% |
|  | A lot of effort | 12 | 23% |
|  | Huge effort | 1 | 2% |
| Ethicality: How fair *is eMindfulness Mode* for people with body image concerns? | Very unfair | 0 | 0% |
|  | Unfair | 0 | 0% |
|  | No opinion | 8 | 15% |
|  | Fair | 36 | 68% |
|  | Very fair | 9 | 17% |
| Perceived effectiveness: *eMindfulness Mode* has improved my body image | Strongly disagree | 0 | 0% |
|  | Disagree | 7 | 13% |
|  | No opinion | 17 | 32% |
|  | Agree | 27 | 51% |
|  | Strongly agree | 2 | 4% |
| Intervention coherence: It is clear to me how *eMindfulness Mode* could help improve body image | Strongly disagree | 0 | 0% |
|  | Disagree | 1 | 2% |
|  | No opinion | 5 | 9% |
|  | Agree | 34 | 64% |
|  | Strongly agree | 13 | 25% |
| Self-efficacy: How confident did you feel about using *eMindfulness Mode*? | Very unconfident | 1 | 2% |
|  | Unconfident | 4 | 8% |
|  | No opinion | 6 | 11% |
|  | Confident | 31 | 58% |
|  | Very confident | 11 | 21% |
| Opportunity costs: Using *eMindfulness Mode* interfered with my other priorities | Strongly disagree | 12 | 23% |
|  | Disagree | 20 | 38% |
|  | No opinion | 10 | 19% |
|  | Agree | 10 | 19% |
|  | Strongly agree | 1 | 2% |
| General acceptability: How acceptable was *eMindfulness Mode* to you? | Completely unacceptable | 0 | 0% |
|  | Unacceptable | 0 | 0% |
|  | No opinion | 9 | 17% |
|  | Acceptable | 20 | 38% |
|  | Completely acceptable | 24 | 45% |
| *Note*. TFA = theoretical framework of acceptability. Response distributions by intervention component were not presented separately due to minimal variation across groups (see Table 1 in the main text). | | | |
